# New Potential Antimalarial Agents: Design, Synthesis and Biological Evaluation of Some Novel Quinoline Derivatives as Antimalarial Agents

**DOI:** 10.3390/molecules21070909

**Published:** 2016-07-14

**Authors:** Ibrahim Ali M. Radini, Tarek M. Y. Elsheikh, Emad M. El-Telbani, Rizk E. Khidre

**Affiliations:** 1 Chemistry Department, Faculty of Science, Jazan University, Jazan 2097, Saudi Arabia; iradini4@gmail.com (I.A.M.R.); dremade1966@yahoo.co.uk (E.M.E.-T.); 2 Biology Department, Faculty of Science, Jazan University, Jazan 2097, Saudi Arabia; telsheikh1964@yahoo.com; 3 Department of Zoology, Faculty of Science, Al-Azhar University, Cairo 11675, Egypt; 4 Chemical Industries Division, National Research Centre, Dokki 12622, Giza, Egypt

**Keywords:** quinoline-4-carbaldehyde, Biginelli reaction, dihydropyrimidines, antimalarial activities, *Plasmodium falciparum*

## Abstract

A novel series of dihydropyrimidines (DHPMs) **4a**–**j**; 2-oxopyran-3-carboxylate **7a,b**; 1-amino-1,2-dihydropyridine-3-carboxylate **8**; and 1,3,4-oxadiazole derivatives **12** with quinolinyl residues have been synthesized in fairly good yields. The structure of the newly synthesized compounds was elucidated on the basis of analytical and spectral analyses. In vitro antimalarial evaluation of the synthesized quinoline derivatives against *Plasmodium falciparum* revealed them to possess moderate to high antimalarial activities, with IC_50_ values ranging from 0.014–5.87 μg/mL. Compounds **4b**,**g**,**i** and **12** showed excellent antimalarial activity against to *Plasmodium falciparum* compared with the antimalarial agent chloroquine (CQ).

## 1. Introduction

**Figure 1:**
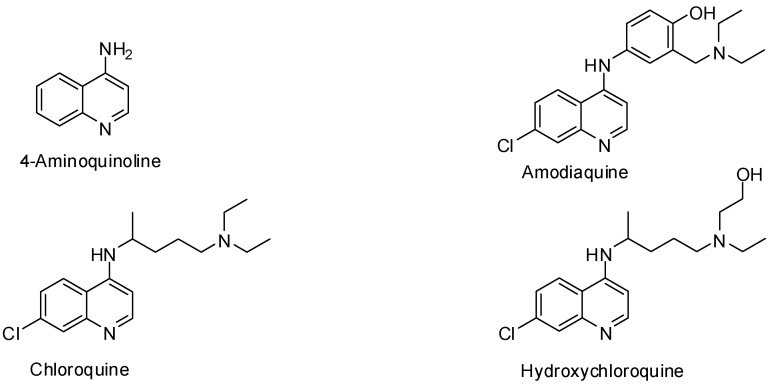
Aminoquinoline and their derivatives as antimalarial agents.

Malaria is one of the principal diseases of the developing countries, particularly in Africa, Asia and South America. According to a World Health Organization (WHO) report, there are between 300 million and 500 million cases of malaria worldwide annually and more than one million people die from that disease, most of them are children under the age of five years [1,2]. Among five typically recognized *Plasmodium* species causing this disease in humans, *Plasmodium falciparum* is responsible for about 95% of worldwide malaria and has a mortality rate of 1%–3%, and *Plasmodium vivax* for most morbidity, additionally representing a reservoir of latent infection that hampers current control and future elimination efforts [3,4,5,6]. Due to the toxic side effects and the risk of developing resistance after prolonged treatment with aminoquinolines and their derivatives (Figure 1) which are nowadays used as antimalarial agents, the growth of and increasing resistance [7,8] of the malaria parasite *Plasmodium falciparum* to known antimalarial agents demands a continuous effort to develop new antimalarial agents especially, as an effective vaccine for malaria is not available.

In addition, quinoline-based fused heterocyclic systems are found to possess potential antimicrobial [9,10], antimalarial [11,12], anti-inflammatory [13,14], antitumor [15], and anti-parasitic activity [16].

Currently there are only limited safe drugs for the treatment of the disease, however, the design of new chemical agents, specifically affecting these targets, could lead to the availability of better drugs to treat malaria. Based on the above information and in continuation with our previous work [11,15,17,18], quinoline-based antimalarials that would not induce resistance, we have designed and prepared several quinolines compounds and screened for their antimalarial activities. Hopefully, these compounds will be active on the CQ-resistant strain FcB1 and could lead to the availability of better drugs to treat malaria.

## 2. Results

### 2.1. Chemistry

**Scheme 1:**
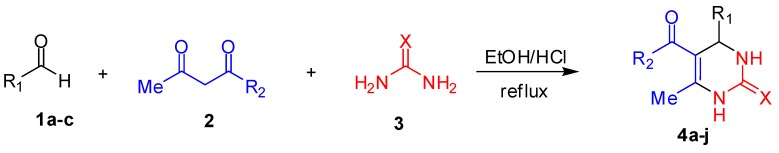
Synthesis of dihydropyridine (DHPMs) **4a**–**j**.

Dihydropyrimidin-2(1*H*)-ones (DHPMs) and their derivatives have received much attention because they are important substructures in both biologically active compounds and several marine alkaloids involving DHPM core units [19,20,21]. A facile three-component Biginelli’s one-pot cyclo-condensation reaction takes place between the quinolinealdehydes namely, tetrazolo-[1,5-*a*]quinoline-4-carbaldehyde (**1a**), 7-methyltetrazolo[1,5-*a*]quinoline-4-carbaldehyde (**1b**) [22,23], 2-oxo-1,2-dihydroquinoline-3-carbaldehyde (**1c**) [24]; ethyl acetoacetate (**2a**) or acetylacetone (**2b**); and (thio)urea **3** in ethanol in the presence of a catalytic amount of hydrochloric acid at reflux temperature to yield dihydropyrimidine (DHPMs) **4a**–**j** in good yield [25,26] (Scheme 1 and Experimental Section).

The structure of products **4a**–**j** has been confirmed by both analytical and spectral analyses. The presence of a single proton at a range of δ = 5.41–5.91 ppm corresponding to H-4 of DHPMs in addition to the two NH groups at δ = 7.13–9.24 ppm and 9.29–11.79 ppm supported the suggested DHPMs structures. Also, molecular weight determination (M_S_) confirmed their structures. (cf. Scheme 1 and Experimental Section).

**Scheme 2:**
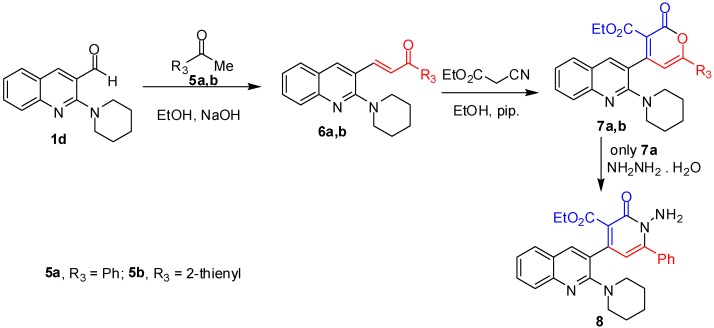
Synthesis of quinolinyl 1,2-dihydropyridine **8**.

The synthetic strategies adopted to obtain the target **8** and **12** are somewhat long and linear with few common intermediates. To this aim, the chalcones derivatives **6a**,**b**, which were prepared by reaction of 2-(piperidin-1-yl)quinoline-3-carbaldehyde (**1d**) [27] with methyl ketones **5a**,**b**, were reacted with ethyl cyanoacetate in ethanol at room temperature to give pyran-3-carboxylate derivatives **7a**,**b** in fairly good yield (Scheme 2). The structures of compounds **7** were established by both analytical and spectral analyses. The IR spectra show two absorption bands at 1690–1682 cm^−1^ and 1743–1736 cm^−1^ for the ester and lactone carbonyl groups, respectively. In addition, the ^1^H-NMR shows the pyran H-5 at δ 7.33–7.39 ppm and other protons in their expected locations. *N*-Nucleophilic addition reaction of hydrazine at the lactonic carbonyl group of **7a**, gave 1,2-dihydropyridine-3-carboxylate derivative **8**. The IR spectra showed absence of the lactonic carbonyl group perilously appeared in the parent **7** and the appearance of new bands at ν 3383, 3182 cm^−1^ due to NH_2_ function and ^1^H-NMR showed a singlet signal at δ 5.41 ppm attributed to amino group.

**Scheme 3:**
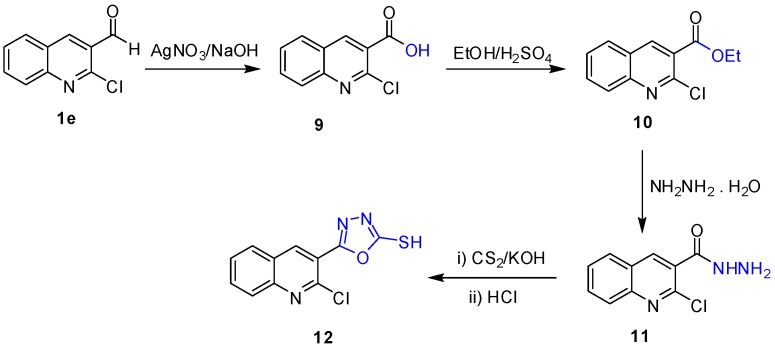
Synthesis of quinoliny 1,3,4-oxadiazole **12**.

2-Chloroquinoline-3-carboxylic acid was prepared by oxidation of **1e** using silver nitrate in the presence of sodium hydroxide [28]. Esterification of the carboxylic acid derivative **9** using absolute ethanol and sulfuric acid afforded the ester derivative **10**, in a good yield, followed by subsequent hydrazinolysis in boiling ethanol to afford 2-chloroquinoline-3-carbohydrazide **11**. The later compound **11** was subjected to react with carbon disulfide in ethanol in the presence of KOH under reflux followed by acidification by using diluted hydrochloric acid to give 5-(2-chloro-quinolin-3-yl)-1,3,4-oxadiazole-2-thiol (**12**). The IR spectrum showed the presence of the absorption band at 2500 cm^−1^ due to S-H function, in addition ^13^C-NMR revealed signal at δ_C_ 164.54 (C2-1,3,4-oxadiazole) ppm indicates that **12** exists in the thiol form (cf. Scheme 3 and Experimental Section).

### 2.2. Antimalarial Evaluation

Seventeen quinoline derivatives were evaluated in vitro against *P. falciparum.* The results of the antimalarial screening are presented in Table 1, Table 2 and Table 3. The basic measurement of antimalarial activity used in this study was the reduction in number of parasitized cells in the test cultures compared to control at 36–48 h of incubation. Compounds exhibiting IC_50_ *P. falciparum* >5 μg/mL was considered inactive. If the IC_50_ is between 0.5 and 5 μg/mL, the compound is classified as moderately active. If the IC_50_ is <0.5 μg/mL, the compound is classified as active.

The percentages of inhibition plasmodial parasite were recorded in Table 1, Table 2 and Table 3. The data revealed that the highest percent of inhibition (100%) was recorded by **4b**, **4g**, **4i**, **6a**, **7b** and **8b** at the concentrations 5.0, 1.25, 2.50, 5.0, 5.0, and 2.50 μg/mL, respectively. Also, **4b**, **4c**, **4g**–**i**, **8a**, **10**, **11** and **12** showed inhibition more than 75% of plasmodial parasite at the concentrations 1.25, 5.0, 0.625, 5.0, 0.625, 2.50, 5.0, 5.0 and 2.50 μg/mL, respectively. At the lowest concentrations 0.312, 0.156 and 0.078 μg/mL, **4b**, **4g**, **4i** showed significant effect against parasite where the inhibition percentage were 43.3% ± 0.89%, 24.4% ± 0.54%, and 34.8% ± 1.16%.

Median inhibitory concentrations (IC_50_) of synthesized compounds against *P. falciparum* in vitro are summarized in Table 3. The IC_50_ values for these compounds were in the range 0.014–15.87 μg/mL. Two compounds exhibited IC_50_ values more than 5 μg/mL against *P. falciparum.* Twelve compounds (**4a**, **4c**, **4f**, **4h**, **4j**, **6a**, **7a**,**b**, **8a**,**b**, **10**, **11**) showed IC_50_ values between 0.5 μg/mL and 5 μg/mL, and considered a moderately active. Four compounds (**4b**, **4j**, **4i** and **12**) were classified as active with IC_50_ (0.46, 0.30, 0.014 and 0.46 μg/mL) compared with chloroquine 0.49 μg/mL.

### 2.3. Structure Activity Relationship

Structure-activity relationship (SAR) revealed that pyrimidine-2-thione moieties incorporating tetrazolo, methyltetrazolo or quinolinone substituents at position 4 along with an acetyl or ester group at position 5 (compounds **4b**, **4g**, **4i**) have greater potency than the corresponding pyrimidine compounds with the same substituents except compound **4d** which has a lowest effect (IC_50_ = 15.87 μg/mL). A combination of the three substituents (quinolinone, acetyl, and methyl) at positions 4, 5, and 6 of the pyrimidine-2-thione has the best activity and greater (IC_50_ = 0.041 μg/mL) than the corresponding pyrimidine, notably **4h** (IC_50_ = 2.21 μg/mL). 2-Chloroquinoline moiety containing a 1,3,4-oxadiazole residue at position 3 (compound **12**) has greater activity (IC_50_ = 0.46 μg/mL) than the corresponding compounds where ethyl ester, or carbohydrazide groups present at position 3 in compounds **10** and **11** (IC_50_ =1.91 μg/mL and 2.21 μg/mL, respectively).

## 3. Experimental Section

### 3.1. General Information

Melting points were determined on digital MFB-595 instrument (Gallenkamp London, UK) using open capillary tubes and are uncorrected. IR spectra were recorded on a FTIR 440 spectrometer (Shimadzu, Tokyo, Japan) using KBr pellets. Mass spectra were obtained on a Qp-2010 plus mass spectrometer (Shimadzu) at 70 eV. ^1^H-NMR and ^13^C-NMR spectra were recorded on a model Ultra Shield-NMR spectrometer (500 MHz or 400 MHz, Bruker, Coventry, UK) in DMSO-*d*_6_ using tetramethylsilane (TMS) as an internal standard at the College of Science, King Khalid University, Saudi Arabia; chemical shifts are reported as δ ppm units. The elemental analyses (% C, H, N) were done at the Microanalytical Center, Cairo University, Cairo, Egypt. Solvents were dried by standard techniques. The monitoring of the progress of all reactions and homogeneity of the synthesized compounds was carried out and was run using thin layer chromatography (TLC) aluminum sheets silica gel 60 F_254_ (Merck, Darmstadt, Germany).

### 3.2. Synthesis

#### 3.2.1. General Procedure for the Synthesis of Dihydropyrimidines (DHPMs) **4a**–**j**

A mixture of the appropriate aromatic aldehyde **1a**–**c** (10 mmol), ethyl acetoacetate (**2a**) (or acetylacetone (**2b**)) (10 mmol), urea (**3a**) (or thiourea (**3b**)) (10 mmol) in ethanol (50 mL) in the presence of a catalytic amount of hydrochloric acid was refluxed for 8–12 h (TLC). The precipitated crude was filtered off, washed with ethanol and recrystallized from acetic acid to give pure crystals of DHPMs **4a**–**j**.

*Ethyl-6-methyl-2-oxo-4-(tetrazolo[1,5-a]quinolin-4-yl)-1,2,3,4-tetrahydropyrimidine-5-carboxylate* (**4a**) Yellow crystals, m.p. 278–280 °C; IR (cm^−1^): ν 3256 and 3122 (2N–H str.), 1725 (C=O), 1693 (C=O), 1610 (C=N), 1556 (C=C); ^1^H-NMR (DMSO-*d*_6_): δ_H_ 0.94 (t, 3H, *J* = 6.6 Hz, CH_3_), 2.29 (s, 3H, CH_3_), 3.99 (q, 2H, *J* = 6.6 Hz, CH_2_), 5.85 (s, 1H, CH), 7.74 (s, 1H, D_2_O exchangeable, NH), 7.79–8.59 (m, 5H, quinoline-H), 9.49 (s, 1H, D_2_O exchangeable, NH) ppm; ^13^C-NMR (DMSO-*d*_6_): δ_C_ 13.88, 18.06, 52.19, 59.07, 95.22, 116.11, 123.56, 128.33, 128.60, 129.33, 129.58, 130.29, 131.29, 146.29, 150.33 (quinoline-C), 151.33 (CONH), 165.01 (C=O, ester) ppm; EI-Ms: *m*/*z* (%): 353 [M^+^ + 1]. Anal. Calcd for C_17_H_16_N_6_O_3_ (352.35.): C, 57.95; H, 4.58; N, 23.85; Found C, 57.80; H, 4.60; N, 23.70.

*Ethyl-6-methyl-4-(tetrazolo[1,5-a]quinolin-4-yl)-2-thioxo-1,2,3,4-tetrahydropyrimidine-5-carboxylate* (**4b**) Yellow crystals, m.p. 263–265 °C; IR (cm^−1^): ν 3275 and 3146 (2NH), 1700 (C=O), 1690 (C=O), 1612 (C=N), 1588 (C=C); ^1^H-NMR (DMSO-*d*_6_): δ_H_ 1.02 (t, 3H, *J* = 6.77 Hz, CH_3_), 2.20 (s, 3H, CH_3_), 4.10 (q, 2H, *J* = 6.77 Hz, CH_2_), 5.80 (s, 1H, CH), 7.74 (s, 1H, D_2_O exchangeable, NH), 7.79–8.59 (m, 5H, quinoline-H), 10.39 (s, 1H, D_2_O exchangeable, NH) ppm; ^13^C-NMR (DMSO-*d*_6_): δ_C_ 13.88, 18.06, 52.19, 59.07, 95.22, 116.11, 123.56, 128.33, 128.60, 129.33, 129.58, 130.29, 131.29, 146.29, 150.33 (quinoline-C), 151.33 (CONH), 165.01 (C=O, ester) ppm; EI-Ms: *m*/*z* (%): 368 [M^+^, 35]; Anal. Calcd for C_17_H_16_N_6_O_2_S (368.41): C, 55.42; H, 4.38; N, 22.81; Found C, 55.60; H, 4.50; N, 22.90.

*5-Acetyl-6-methyl-4-(tetrazolo[1,5-a]quinolin-4-yl)-3,4-dihydropyrimidin-2(1H)-one* (**4c**) Yellow crystals, m.p. ˃300 °C; IR (cm^−1^): ν 3350 and 3294 (2NH), 1687 (C=O),1651 (C=O), 1622 (C=N), 1565 (C=C); ^1^H-NMR (DMSO-*d*_6_): δ_H_ 2.18 (s, 3H, CH_3_), 2.30 (s, 3H, CH_3_), 5.65 (s, 1H, CH), 7.20–7.80 (m, 5H, quinoline-H), 8.01 (s, 1H, D_2_O exchangeable, NH), 9.29 (s,1H, D_2_O exchangeable, NH) ppm; ^13^C-NMR (DMSO-*d*_6_): δ_C_ 17.35, 29.88, 49.50, 106.95, 116.87, 118.89, 121.94, 128.11, 130.22, 133.04, 134.65, 138.09, 149.12, 150.27 (quinoline-C), 152.26 (C=O), 193.22 (C=O) ppm; EI-Ms: *m*/*z* (%): 322 [M^+^, 23]; Anal. Calcd for C_16_H_14_N_6_O_2_ (322.32): C, 59.62; H, 4.38; N, 26.06; Found C, 59.80; H, 4.20; N, 26.20.

*1-(6-Methyl-4-(tetrazolo[1,5-a]quinolin-4-yl)-2-thiooxo-1,2,3,4-tetrahydropyrimidin-5-yl)ethanone* (**4d**) Yellow crystals, m.p. 280–282 °C; IR (cm^−1^): ν 3373, 3172 (2NH), 1685 (C=O), 1628 (C=N), 1588 (C=C); ^1^H-NMR (DMSO-*d*_6_): δ_H_ 2.23 (s, 3H, CH_3_), 2.39 (s, 3H, CH_3_), 5.57 (s, 1H, CH), 7.18–7.77 (m, 5H, quinoline-H), 7.95 (s, 1H, D_2_O exchangeable, NH), 9.44 (s,1H, D_2_O exchangeable, NH) ppm; ^13^C-NMR (DMSO-*d*_6_): δ_C_ 17.88, 29.70, 49.67, 106.17, 114.83, 118.71, 127.40, 128.17, 130.40, 132.46, 135.76, 138.23, 146.39, 151.22 (quinoline-C), 161.98 (C=O), 174.60 (C=S) ppm; EI-Ms: *m*/*z* (%): 338 [M^+^, 25]; Anal. Calcd for C_16_H_14_N_6_OS (338.39): C, 56.79; H, 4.17; N, 24.84; Found C, 56.80; H, 4.30; N, 24.70.

*Ethyl-6-methyl-4-(7-methyltetrazolo[1,5-a]quinolin-4-yl)-2-oxo-1,2,3,4-tetrahydropyrimidine-5-carboxylate* (**4e**) Yellow crystals, m.p. 293 °C; IR (cm^−1^): ν 3325, 3271 (2NH), 1720 (CO, ester), 1675 (C=O), 1610 (C=N), 1596 (C=C); ^1^H-NMR (DMSO-*d*_6_): δ_H_ 1.19 (t, 3H, *J* = 6.50 Hz, CH_3_), 2.33 (s, 3H, CH_3_), 2.55 (s, 3H, CH_3_), 4.20 (q, 2H, *J* = 6.50 Hz, CH_2_), 5.91 (s, 1H, CH), 7.13 (s, 1H, D_2_O exchangeable, NH), 7.77 (d, 1H, *J* = 8.5 Hz, quinoline-H), 7.97 (d, 1H, *J* = 8.5 Hz, quinoline-H), 8.19 (s, 1H, quinoline-H), 8.46 (s, 1H, quinoline-H), 9.55 (s, 1H, D_2_O exchangeable, NH); ^13^C-NMR (DMSO-*d*_6_): δ_C_ 13.91, 18.03, 20.84, 52.11, 62.49, 59.07, 95.22, 123.63, 127.45, 128.45, 128.96, 129.31, 129.51, 132.62, 138.10, 145.70 (quinoline-C), 151.38 (CONH), 157.66 (C=O, ester) ppm; EI-Ms: *m*/*z* (%): 367 [M^+^ + 1, 15], Anal. Calcd for C_18_H_18_N_6_O_3_ (366.37): C, 59.01; H, 4.95; N, 22.94; Found C, 59.20; H, 4.90; N, 22.90.

*Ethyl-6-methyl-4-(7-methyltetrazolo[1,5-a]quinolin-4-yl)-2-thioxo-1,2,3,4-tetrahydropyrimidine-5-carboxylate* (**4f**) Yellow crystals, m.p. 289 °C; IR (cm^−1^): ν 3325, 3271 (2NH), 1710 (C=O, ester), 1683 (C=O), 1624 (C=N), 1596 (C=C); ^1^H-NMR (DMSO-*d*_6_): δ_H_ 1.24 (t, 3H, *J* = 6.55 Hz, CH_3_), 2.33 (s, 3H, CH_3_), 2.55 (s, 3H, CH_3_), 4.10 (q, 2H, *J* = 6.55 Hz, CH_2_), 5.85 (s, 1H, CH), 7.80 (s, 1H, D_2_O exchangeable, NH), 7.82 (d, 1H, *J* = 8.7 Hz, quinoline-H), 8.01 (d, 1H, *J* = 8.7 Hz, quinoline-H), 8.11 (s,1H, quinoline-H), 8.50 (s, 1H, quinoline-H), 9.59 (s, 1H, D_2_O exchangeable, NH); ^13^C-NMR (DMSO-*d*_6_): δ_C_ 13.81, 17.30, 20.84, 51.98, 59.42, 115.97, 123.67, 127.40, 128.66, 128.96, 129.06, 129.22, 130.84, 132.82, 138.27, 146.59 (quinoline-C), 157.66 (C=O, ester), 174.55 (C=S) ppm; EI-Ms: *m*/*z* (%): 382 [M^+^, 23]; Anal. Calcd for C_18_H_18_N_6_O_2_S (382.44.): C, 56.53; H, 4.74; N, 21.97; Found C, 56.50; H, 4.80; N, 21.80.

*1-(6-Methyl-4-(7-methyltetrazolo[1,5-a]quinolin-4-yl)-2-thioxo-1,2,3,4-tetrahydropyrimidine-5-ethanone* (**4g**) Yellow crystals, m.p. 280–282 °C; IR (cm^−1^): ν 3290, 3215 (2NH), 1700 (C=O), 1655 (C=O), 1614 (C=N), 1586 (C=C); ^1^H-NMR (DMSO-*d*_6_): δ_H_ 2.33 (s, 3H, CH_3_), 2.40 (s, 3H, CH_3_), 2.60 (s, 3H, CH_3_), 5.77 (s, 1H, CH), 7.49 (s, 1H, D_2_O exchangeable, NH), 7.77 (d, 1H, *J* = 9 Hz, quinoline-H), 7.99 (d, 1H, *J* = 9 Hz, quinoline-H), 8.10 (s, 1H, quinoline-H), 8.50 (s, 1H, quinoline-H), 9.44 (s, 1H, D_2_O exchangeable, NH); ^13^C-NMR (DMSO-*d*_6_): δ_C_ 17.81, 20.84, 29.18, 50.98, 106.17, 114.83, 118.71, 128.17, 130.40, 132.46, 135.76, 136.4, 138.23, 146.39, 151.22 (quinoline-C), 174.60 (C=S), 192.20 (C=O) ppm; EI-Ms: *m*/*z* (%): 352 [M^+^, 45]; Anal. Calcd for C_17_H_16_N_6_OS (352.41.): C, 57.94; H, 4.58; N, 23.85; Found C, 57.80; H, 4.70; N, 23.80.

*3-(5-Acetyl-6-methyl-2-oxo-1,2,3,4-tetrahydropyridin-4yl)quinolin-2(1H)-one* (**4h**) Yellow crystals, m.p. ˃300 °C; IR (cm^−1^): ν 3301, 3224, 3180 (3NH), 1700 (C=O), 1649 (C=O), 1618 (C=N), 1583 (C=C); ^1^H-NMR (DMSO-*d*_6_): δ_H_ 2.16 (s, 3H, CH_3_), 2.35 (s, 3H, CH_3_), 5.44 (s, 1H, CH), 7.16–8.04 (m, 4H, quinoline-H), 8.32 (s, 1H, quinoline-H), 9.24 (s,1H, D_2_O exchangeable, NH), 11.97 (s, 1H, D_2_O exchangeable, NH), 12.31 (s, 1H, D_2_O exchangeable, NH); ^13^C-NMR (DMSO-*d*_6_): δ_C_ 18.35, 29.91, 49.25, 106.95, 114.87, 118.89, 121.94, 128.11, 130.22, 133.04, 134.65, 138.09, 149.12 (quinoline-C), 152.26 (C=O), 161.32 (C=O), 194.22 (C=O) ppm; EI-Ms: *m*/*z* (%): 297 [M^+^]; Anal. Calcd for C_16_H_15_N_3_O_3_ (297.31.): C, 64.64; H, 5.09; N, 14.13; Found C, 64.50; H, 5.20; N, 14.20.

*3-(5-Acetyl-6-methyl-2-thioxo-1,2,3,4-tetrahydropyrimidin-4yl)quinolin-2(1H)-one* (**4i**) Yellow crystals, m.p. ˃300 °C; IR (cm^−1^): ν 3299, 3234, 3172 (3NH), 1651 (C=O), 1610 (C=N), 1590 (C=C); ^1^H-NMR (DMSO-*d*_6_): δ_H_ 2.20 (s, 3H, CH_3_), 2.30 (s, 3H, CH_3_), 5.50 (s, 1H, CH), 7.16–7.95 (m, 4H, quinoline-H), 8.20 (s, 1H, quinoline-H), 9.11 (s,1H, D_2_O exchangeable, NH), 11.82 (s, 1H, D_2_O exchangeable, NH), 12.20 (s, 1H, D_2_O exchangeable, NH); ^13^C-NMR (DMSO-*d*_6_): δ_C_ 17.19, 49.78, 59.42, 104.66, 114.83, 118.71, 127.40, 128.17, 129.23, 130.40, 132.46, 135.76, 138.23, 146.39 (quinoline-C), 164.67 (C=O), 174.50 (C=S) ppm; EI-Ms: *m*/*z* (%): 313 [M^+^]; Anal. Calcd for C_16_H_15_N_3_O_2_S (313.37): C, 61.37; H, 4.82; N, 13.41; Found C, 61.20; H, 4.70; N, 13.20.

*Ethyl-6-methyl-4-(2-oxo-1,2-dihydroquinolin-3-yl)-2-thioxo-1,2,3,4-tetrahydropyrimidine-5-carboxylate* (**4j**) Yellow crystals, m.p. ˃300 °C; IR (cm^−1^): ν 3377, 3224, 3186 (3NH), 1780 (C=O, ester), 1660 (C=O), 16118 (C=N), 1566 (C=C); ^1^H-NMR (DMSO-*d*_6_): δ_H_ 1.08 (t, 3H, *J* = 7 Hz, CH_3_), 2.36 (s, 3H, CH_3_), 4.01 (q, 2H, *J* = 7 Hz, CH_2_), 5.41 (s, 1H, CH), 7.17 (dd, 1H, *J* = 1 Hz, *J* = 8 Hz, quinoline-H), 7.49 (dd, 1H, *J* = 1.35 Hz, *J* = 7.25 Hz, quinoline-H), 7.53 (dd, 1H, *J* = 1.5 Hz, *J* = 9 Hz, quinoline-H), 7.71 (dd, 1H, *J* = 2 Hz, *J* = 8.2 Hz), 7.72 ( s, 1H, quinoline-H), 9.01 (s, 1H, D_2_O exchangeable, NH), 10.33 (s, 1H, D_2_O exchangeable, NH), 11.90 (s, 1H, D_2_O exchangeable, NH); ^13^C-NMR (DMSO-*d*_6_): δ_C_ 14.01, 17.19, 49.78, 59.42, 97.97, 114.83, 118.71, 127.40, 128.17, 130.40, 132.46, 135.76, 138.23, 146.39 (quinoline-C), 160.80 (C=O, ester) ,164.98 (C=O), 174.64 (C=S) ppm; EI-Ms: *m*/*z* (%): 343.95 [M^+^, 35], Anal. Calcd for C_17_H_17_N_3_O_3_S (343.40.): C, 59.46; H, 4.99; N, 12.24; Found C, 59.50; H, 4.80; N, 12.20.

#### 3.2.2. General Procedure for the Synthesis of Chalcones **6a**,**b**

To a stirred solution of acetophenone (**5a**) or 2-acetylthiophene (**5b**) (10 mmol) in alcoholic NaOH solution (5%, 25 mL) at 0–5 °C a solution of 2-(piperidin-1-yl) quinoline-3-carbaldehyde (**1e**) (2.40 g, 10 mmol) was added gradually. Stirring was continued for 24 h at r.t. the resulting precipitate was filtrated, washed with EtOH (10 mL), and dried, and crystallized from ethanol.

*1-Phen.yl-3-[2-(piperidin-1-yl)quinolin-3-yl]prop-2-en-1-one* (**6a**). Yellow crystals, m.p. 127–129 °C, IR (cm^−1^): ν 1654 (C=O), 1585 (C=C); ^1^H-NMR (CDCl_3_): δ_H_ 1.62–1.77 (m, 6H, CH_2_-piperidinyl), 3.30–3.34 (m, 4H, CH_2_-piperidinyl), 6.94 (d, 1H, *J* = 18.90 Hz, CH=CH), 7.40–7.79 (m, 10H, quinoline-H & Ar-H & CH=CH), 8.14 (s, 1H, quinoline-H); EI-Ms: *m*/*z* (%): 342 [M^+^, 60]; Anal. Calcd for C_23_H_22_N_2_O (342.43.): C, 80.67; H, 6.48; N, 8.18; Found C, 80.50; H, 6.60; N, 8.20.

*3-[2-(Piperidin-1-yl)quinolin-3-yl]-1-thiophene-2-yl)prop-2-en-1-one* (**6b**). Yellow crystals, m.p. 162–164 °C (EtOH); IR (cm^−1^): ν 1649 (C=O), 1595 (C=C); ^1^H-NMR (CDCl_3_): δ_H_ 1.66–1.80 (m, 6H, CH_2_-piperidinyl), 3.34–3.36 (m, 4H, CH_2_-piperidinyl), 7.19–7.92 (m, 8H, quinoline-H , thiophene-H, CH=CH), 8.02 (d, 1H, *J* = 19.2 Hz, CH=CH), 8.21 (s, 1H, quinoline-H); ^13^C-NMR (CDCl_3_): δ_C_ 24.59, 25.88, 51.83, 122.17, 122.94, 124.32, 127.36, 128.31, 128.38, 130.49, 131.84.132.19, 132.95, 133.99, 137.26, 141.80, 145.48, 160.61(thiophene-C & quinoline-C), 181.97 (C=O) ppm; EI-Ms: *m*/*z* (%): 348 [M^+^, 74], Anal. Calcd for C_21_H_20_N_2_OS (348.46.): C, 72.38; H, 5.79; N, 8.04; Found C, 72.40; H, 5.70; N, 8.10.

#### 3.2.3. General Procedure for the Synthesis of **7a**,**b**

A mixture of **6a**,**b** (10 mmol) and ethyl cyanoacetate (1.13 mL, 10 mmole) in absolute ethanol (20 mL) in the presence of a few drops of piperidine was stirred at room temperature for 6 h. The solid formed was filtered off, washed with ethanol, dried and crystallized from acetic acid.

*Ethyl-2-oxo-6-phenyl-4-[2-(piperidin-1-yl)quinolin-3-yl]-2H-pyran-3-carboxylate* (**7a**). Colorless crystals, m.p. 174–176 °C; IR (cm^−1^): ν 1736 (C=O), 1681 (C=O), 1595 (C=C); ^1^H-NMR (CDCl_3_): δ_H_ 1.38 (t, 3H, *J* = 7.5 Hz, CH_3_), 1.69–1.89 (m, 6H, CH_2_-piperidinyl), 3.53–3.57 (m, 4H, CH_2_-piperidinyl), 4.35 (q, 2H, *J* = 7.5 Hz, CH_2_), 7.39 (s, 1H, pyran H-5), 7.52–7.93 (m, 9H, Ar-H & quinoline-H), 8.06 (s, 1H, quinoline-H); ^13^C-NMR (CDCl_3_): δ_C_ 14.05, 24.45, 26.33, 52.66, 62.68, 115.20, 116.09, 125.17, 125.75, 127.38, 127.56, 127.72, 128.07, 128.17, 128.31, 128.88, 129.47, 133.74, 133.81, 134.74, 136.21, 136.33, 146.71, 161.52 (thiophene-C & quinoline-C), 165.56 (C=O), 196.49 (CO, ester) ppm; EI-Ms: *m*/*z* (%): 356 [M^+^ + 2, 100]; Anal. Calcd for C_28_H_26_N_2_O_4_ (454.52.): C, 73.99; H, 5.77; N, 6.16; Found C, 73.90; H, 5.70; N, 6.10.

*Ethyl-2-oxo-4-[2-(piperidin-1-yl)quinolin-3yl]-6-(thiophen-2-yl)-2H-pyran-3-carboxylate* (**7b**). Colorless crystals, m.p. 154–156 °C; IR (cm^−1^): ν 1743 (CO), 1662 (C=O), 1596 (C=C). ^1^H-NMR (CDCl_3_): δ_H_ 1.34 (t, 3H, *J* = 6.95 Hz, CH_3_), 1.65–1.80 (m, 6H, CH_2_-piperidinyl), 3.50–3.59 (m, 4H, CH_2_-piperidinyl), 4.30 (q, 2H, *J* = 6.95 Hz, CH_2_), 7.33 (s, 1H, pyran H-5), 7.40–8.06 (m, 7H, quinoline-H & thiophene-H), 8.20 (s, 1H, quinoline-H); EI-Ms: *m*/*z* (%): 461 [M^+^ + 1, 78], Anal. Calcd for C_26_H_24_N_2_O_4_S (460.54.): C, 67.81; H, 5.25; N, 6.08; Found C, 67.80; H, 5.400; N, 6.20.

*Ethyl-1-amino-2-oxo-6-phenyl-4-(2-(piperidin-1-yl)quinolin-3-yl)-1,2-dihydropyridine-3-carboxylate* (**8**). A mixture of **7b** (4.6 g, 10 mmol) and hydrazine hydrate (10 mmol) in absolute ethanol (20 mL) was stirred at refluxed temperature for 8 h. The formed solid was filtered off, washed with ethanol, dried and crystallized from acetic acid to give as colorless crystals, m.p. 184–186 °C, (80%, yield) IR (cm^−1^): ν 3383, 3182 (NH_2_), 1690 (C=O), 1656 (C=O), 1588 (C=C); ^1^H-NMR (DMSO-*d*_6_): δ_H_ 1.19 (t, 3H, *J* = 6.67 Hz, CH_3_), 1.73–1.84 (m, 6H, CH_2_-piperidinyl), 3.33–3.43 (m, 4H, CH_2_-piperidinyl), 4.44 (q, 2H, *J* = 6.67 Hz, CH_2_), 5.41 (s, 2H, D_2_O exchangeable, NH_2_), 6.70 (s, 1H, pyran H-4), 7.59–7.92 (m, 9H, Ar-H & quinoline-H), 8.79 (s, 1H, quinoline-H); ^13^C-NMR (DMSO-*d*_6_): δ_C_ 18.52, 23.42, 26.34, 52.67, 56.00, 125.47, 126.77, 126.97, 127.01, 127.27, 127.73,127.87, 127.93, 128.79, 128.83, 129.88, 131.11, 132,19, 134.28, 136.09 ,137.74, 139.34, 144.87, 145.40 (Ar-C & quinoline-C), 150.26 (C=O), 165.66 (C=O) ppm; EI-Ms: *m*/*z* (%): 468 [M^+^, 25], Anal. Calcd for C_28_H_28_N_4_O_3_ (468.54): C, 71.78; H, 6.02; N, 11.96; Found C, 71.57; H, 6; N, 11.98.

*Ethyl-2-chloroquinolin-3- carboxylate* (**10**). To a solution of 2-chloroquinoline-3-carboxylic acid (**9**, 2.07 g, 10 mmol) in absolute ethanol (50 mL), 5 drops of conc. H_2_SO_4_ were added and the reaction mixture was heated under reflux for 8 h. The solution was poured onto crushed ice water; the precipitate was filtered, washed with water, dried and recrystallized from ethanol to afford **10**. White crystals, 82% yield, m.p. 295–297 °C; IR (cm^−1^): ν 1716 (C=O), 1611 (C=N), 1596 (C=C); ^1^H-NMR (CDCl_3_): δ_H_ 1.3 (t, 3H, *J* = 7.25 Hz, CH_3_), 4.34 (q, 2H, *J* = 7.25 Hz, CH_2_), 7.38–8.12 (m, 4H, quinoline-H), 8.54 (s, 1H, quinoline-H) ppm; EI-Ms: *m*/*z* (%): 235 [M^+^, 100], 237 [M^+^ + 2, 33]; Anal. Calcd for C_12_H_10_ClNO_2_ (235.67): C, 61.16; H, 4.28; N, 5.94; Found C, 61.30; H, 4.40; N, 5.80.

*2-Chloroquinoline-3-carbohydrazide* (**11**). A solution of ester derivative **10** (2.35 g, 10 mmol,) and hydrazine hydrate (10 mL) in absolute ethanol (50 mL) was refluxed for 12 h. The solution was cooled to room temperature, poured onto cooled water, the resulting precipitate was filtered, washed with water, (3 × 20 mL) dried and recrystallized from ethanol to give **11** as a white crystals in 76% yield, m.p. ˃300 °C; IR (cm^−1^): ν 3409, 3309 (NH_2_), 3135 (NH), 1652 (C=O), 1611 (C=N), 1585 (C=C), ^1^H-NMR (CDCl_3_): δ_H_ 4.60 (s, brs., 2H, D_2_O exchangeable, NH_2_), 7.48–7.54 (dd, 1H, *J* = 1.60 Hz, *J* = 8.99 Hz, quinoline-H), 7.70–7.75 (m, 1H, quinoline-H), 8.01–8.06 (dd, 1H, *J* = 2.1 Hz, *J* = 8.6 Hz, quinoline-H), 8.85 (s, 1H, quinoline-H), 9.25 (s, 1H, D_2_O exchangeable, NH) ppm; EI-Ms: *m*/*z* (%): 223 [M^+^ + 2, 26], 221 [M^+^, 9]; Anal. Calcd for C_10_H_8_ClN_3_O (221.64): C, 54.19; H, 3.64; N, 18.96; Found C, 54.30; H, 3.50; N, 18.80.

*5-(2-Chloroquinolin-3-yl)-1,3,4-oxadiazole-2)-thiol* (**12**). To a solution of **11** (2.21 g, 10 mmol) in ethanol (10 mL) was added carbon disulfide (50 mmol) and potassium hydroxide (0.65 g, 10 mmol) at 0 °C. The resulting solution was refluxed for 4 h. The solvent was evaporated and the residue dissolved in water and acidified with a diluted solution of HCl. The resulting precipitate was filtered, washed with water, dried and recrystallized from ethanol to afford compound **12**. Yield 78%, Colorless crystals, m.p. 278–280 °C, IR (cm^−1^): 2500 cm^−1^ (-SH, stretching), 1612 (C=N), 1598 (C=C); ^1^H-NMR (DMSO-*d*_6_): δ_H_ 7.38–7.42 (dd, 1H, *J* = 2.1 Hz, *J* = 8.6 Hz, quinoline-H), 7.505–7.522 (d, 1H, *J* = 8 Hz, quinoline-H), 7.75–7.79 (dd, 1H, *J* = 1.5 Hz, *J* = 7 Hz quinoline-H), 8.03–8.04 (dd, 1H, *J* = 1.5 Hz, *J* = 8 Hz, quinoline-H), 8.96 (s, 1H, quinoline-H) 13.4 (s, 1H, SH); ^13^C-NMR (DMSO-*d*_6_): δ_C_ 115.98, 116.14, 116.30, 117.56, 119.18, 123.67, 123.83, 123.99, 130.17, 130.33, 133.85, 134.01, 139.38, 146.36, 146.52, 163.94, 164.54 (C2-1,3,4-oxadiazole) ppm; EI-Ms: *m*/*z* (%): 265 [M^+^ + 2, 11], 263 [M^+^, 33]; Anal. Calcd for C_11_H_6_ClN_3_OS (263.70): C, 50.10; H, 2.29; N, 15.39; Found C, 50.30; H, 2.40; N, 15.20.

### 3.3. Anti-Plasmodial Assay

Blood sample infected with the malaria parasite *P. falciparum* strain was obtained from King Fahd Hospital in Jazan. Parasitemia was measured by examining 1500 red cells with microscopic inspection of Giemsa-stained thin blood smears and is reported as the percent of parasitized erythrocytes. The parasitisma in sample was 3.3%. In vitro experiments were conducted to measure inhibition of parasite growth after incubation of human parasitized red blood cells in media of Roswell Park Memorial Institute (RPMI 1640) and fetal calf serum. Briefly, this procedure involved preparing stock solutions of each compound in dimethyl sulfoxide (DMSO). The stock solutions were diluted to provide test solutions having concentrations in the range 0.078–5.0 μg/mL. Test solutions were transferred to 96-well test plates containing parasitized red blood cells with 3.3% parasitaemia. Each sample was evaluated in triplicate and the test plate was incubated for 48 h at 37 °C. After incubation, quantification of parasites was achieved by optical microscopy on blood smears from each well [29]. The blood films were fixed with methanol alcohol and transferred into a stand and immersed in Giemsa’s stain for 30 min, then washed with tape water and kept to dry until microscopic examination. The ring and schizont forms were counted. Inhibition percentage was calculated using the following formula: Parasite number of control well-Parasite number of treated well/Parasite number of control well ×100.

### 3.4. Statistical Analysis

Statistical analysis of the data was carried out according to the method of Lentner [30]. IC_50_ values were calculated using multiple linear regressions [31].

## 4. Conclusions

A series of new quinoline derivatives has been synthesized starting from tetrazoloquinoline-3-carbaldehyde, 2-oxo-1,2-dihydroquinoline-3-carbaldehyde, 2-chloroquinoline-3-carbaldehyde, and 2-(piperidin-1-yl)quinoline-3-carbaldehyde. In vitro antimalarial evaluation of the synthesized compounds showed moderate to high antimalarial activities, with IC_50_ values ranging from 0.014–15.87 μg/mL. The presence of acetyl groups along with 2-thiooxo-1,2,3,4-tetrahydropyrimidine and 1,3,5-oxadiazole residues incorporating quinoline moieties is responsible for increasing the antimalarial activity compared with classical antimalarial agents (chloroquine).

## Figures and Tables

**Table:** 

Compound	R_1_	R_2_	X	Yield %	Compound	R_1_	R_2_	X	Yield %
**4a**	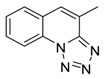	OEt	O	89	**4f**	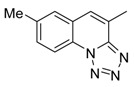	OEt	S	88
**4b**	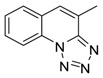	OEt	S	86	**4g**	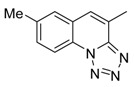	Me	S	86
**4c**	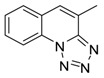	Me	O	87	**4h**	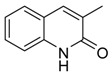	Me	O	85
**4d**	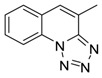	Me	S	85	**4i**	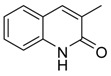	Me	S	89
**4e**	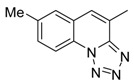	OEt	O	88	**4j**	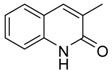	OEt	S	85

**Table 1:** % Parasite inhibition of *Plasmodium falciparum* by some quinolinyl derivatives **4**–**12**.

Compound	% Mean Parasite Inhibition Per 1500 RBC							
	Concentrations (μg/mL)							
	0.625		1.25		2.5		5	
	Range	Mean ± SE	Range	Mean ± SE	Range	Mean ± SE	Range	Mean ± SE
**4a**	2.0–7.8	5.7 ± 1.85	8.2–18.2	14.1 ± 3.02	31.3–41.8	36.6 ± 3.03	47.1–53.1	51.0 ± 1.93
**4c**	14.2–15.6	14.7 ± 0.42	26.5–36.3	35.3 ± 3.09	54.5–62.7	57.4 ± 2.63	85.7–92.1	89.5 ± 1.96
**4d**	2.0–5.4	3.7 ± 0.98	0.0–3.6	1.8 ± 1.03	6.1–7.8	7.1 ± 0.49	14.3–18.1	16.1 ± 1.11
**4e**	0.0–2.0	1.3 ± 0.63	2.0–7.2	4.4 ± 1.51	13.7–14.2	14.1 ± 0.23	24.4–35.2	29.5 ± 3.12
**4f**	9.8–12.2	10.9 ± 0.69	27.4–29.1	28.3 ± 0.49	32.7–43.1	37.5 ± 3.02	69.1–78.4	72.9 ± 2.79
**4h**	20.4–23.6	21.8 ± 0.93	24.5–35.2	31.4 ± 3.05	69.1–75.5	72.3 ± 1.84	80–83.7	82.0 ± 1.07
**4j**	1.9–7.2	3.7 ± 1.75	0.0–5.4	2.5 ± 1.57	19.6–20.4	20.0 ± 0.23	49–56.8	51.6 ± 2.58
**6b**	42.8–43.6	43.2 ± 0.23	52.7–62.7	56.2 ± 3.26	55.1–69.1	61.0 ± 4.18	100–100	100 ± 00
**7a**	27.4–29.1	29.1 ± 0.92	37.3–45.5	42.6 ± 2.63	64.7–67.3	66.4 ± 0.86	66.7–73.5	71.0 ± 2.14
**7b**	34.5–35.3	34.8 ± 0.25	54.5–55.1	54.8 ± 0.17	78.4–89.1	83.0 ± 3.17	100–100	100 ± 00
**8**	35.3–50.1	42.3 ± 4.3	63.3–76.4	70.1 ± 3.8	100–100	100 ± 00	-	-
**10**	11.8–25.5	17.2 ± 4.21	43.1–60	51.4 ± 4.88	67.3–74.5	70.3 ± 2.16	84.3–84.3	84.3 ± 00
**11**	5.9–18.2	12.8 ± 3.62	39.2–49	44.6 ± 2.86	63.2–74.5	70.1 ± 350	75.5–83.6	80.0 ± 2.51
**12**	35.3–45.4	41.8 ± 3.27	59.2–70.9	64.3 ± 3.46	74.5–78.2	76.7 ± 1.13	86.3–91.8	89.7 ± 1.70
**CQ**	59.9–63.8	61.8 ± 1.13	75.9–78.1	76.7 ± 0.67	100–100	100 ± 0.0	-	-

**Table 2:** % Parasite inhibition of *Plasmodium falciparum* (% Mean Parasite Inhibition per 1500 RBC) of compounds **4b**,**g**,**i**.

Concentrations (μg/mL)		Compound			
		4b	4g	4i	CQ
**0.078**	**Range**	NP	NP	32.7–36.7	NP
	**Mean ± SE**	NP	NP	34.8 ± 1.16	NP
**0.156**	**Range**	NP	23.5–25.4	NP	28.9–33.7
	**Mean ± SE**	NP	24.4 ± 0.54	NP	31.8 ± 1.44
**0.312**	**Range**	41.8–44.9	56.3–61.2	63.6–71.4	43.3–46.4
	**Mean ± SE**	43.3 ± 0.89	58.7 ± 1.41	67.8 ± 2.28	44.9 ± 0.90
**0.625**	**Range**	55.1–70.1	82.3–89.7	74.5–86.2	59.9–63.8
	**Mean ± SE**	63.3 ± 4.38	87.0 ± 2.35	79.4 ± 3.5	61.8 ± 1.13
**1.25**	**Range**	73.5–82.3	100–100	85.7–94.1	75.9–78.1
	**Mean ± SE**	76.7 ± 2.78	100 ± 0.0	89.9 ± 2.59	76.7 ± 0.67
**2.5**	**Range**	89.1–95.9	-	100–100	100–100
	**Mean ± SE**	91.7 ± 2.11	-	100 ± 0.0	100 ± 0.0
**5**	**Range**	100–100	-	-	-
	**Mean ± SE**	100 ± 0.0	-	-	-

NP = Not performed.

**Table 3:** IC_50_ values of some derivatives compound of chloroquine against *Plasmodium falciparum*.

Compound	IC_50_ (μg/mL)	Slope	R^2^
**4a**	4.59	10.32	0.929
**4b**	0.46	10.43	0.772
**4c**	2.39	16.28	0.968
**4d**	15.87	3.17	0.932
**4e**	8.10	6.55	0.997
**4f**	3.29	13.34	0.977
**4g**	0.30	61.62	0.791
**4h**	2.21	14.0	0.831
**4i**	0.014	23.48	0.733
**4j**	4.96	11.67	0.975
**6a**	1.13	12.41	0.965
**7a**	2.09	9.11	0.790
**7b**	1.06	14.14	0.889
**8**	0.76	29.79	0.971
**10**	1.91	13.27	0.782
**11**	2.21	13.59	0.767
**12**	0.46	9.62	0.834
**CQ**	0.49	27.01	0.927
